# Systemic Perturbations of Key Metabolites in Diabetic Rats During the Evolution of Diabetes Studied by Urine Metabonomics

**DOI:** 10.1371/journal.pone.0060409

**Published:** 2013-04-03

**Authors:** Mimi Guan, Liyun Xie, Chengfeng Diao, Na Wang, Wenyi Hu, Yongquan Zheng, Litai Jin, Zhihan Yan, Hongchang Gao

**Affiliations:** 1 School of Pharmacy, Wenzhou Medical College, Wenzhou, China; 2 Radiology Department of the Second Affiliated Hospital, Wenzhou Medical College, Wenzhou, China; Broad Institute of Harvard and MIT, United States of America

## Abstract

**Background:**

Elucidation of metabolic profiles during diabetes progression helps understand the pathogenesis of diabetes mellitus. In this study, urine metabonomics was used to identify time-related metabolic changes that occur during the development of diabetes mellitus and characterize the biochemical process of diabetes on a systemic, metabolic level.

**Methodology/Principal Findings:**

Urine samples were collected from diabetic rats and age-matched controls at different time points: 1, 5, 10, and 15 weeks after diabetes modeling. ^1^H nuclear magnetic resonance (^1^H NMR) spectra of the urine samples were obtained and analyzed by multivariate data analysis and quantitative statistical analysis. The metabolic patterns of diabetic groups are separated from the controls at each time point, suggesting that the metabolic profiles of diabetic rats were markedly different from the controls. Moreover, the samples from the diabetic 1-wk group are closely associated, whereas those of the diabetic 15-wk group are scattered, suggesting that the presence of various of complications contributes significantly to the pathogenesis of diabetes. Quantitative analysis indicated that urinary metabolites related to energy metabolism, tricarboxylic acid (TCA) cycle, and methylamine metabolism are involved in the evolution of diabetes.

**Conclusions/Significance:**

The results highlighted that the numbers of metabolic changes were related to diabetes progression, and the perturbed metabolites represent potential metabolic biomarkers and provide clues that can elucidate the mechanisms underlying the generation and development of diabetes as well as its complication.

## Introduction

Diabetes mellitus is a complex metabolic disorder characterized by chronic hyperglycemia, hypoinsulinemia, and ketosis [1]. This condition has become a major healthcare problem because of the rapid increase in the incidence of diabetes and its debilitating complications [2]. Patients with type 1 diabetes are at constant risk for developing a series of morbid and often mortal complications including renal failure, proliferative retinopathy leading to blindness, and neuropathic syndromes [3]. The streptozotocin (STZ)-induced diabetes in a rat model is a T cell-mediated autoimmune disease associated with a marked decrease in insulin levels [4], exhibiting metabolic characteristics similar to those of type 1 diabetes in humans [5].

As a systemic metabolic disease, diabetes mellitus affects multiple organs and tissues throughout the body [6]. Hyperglycemia can have overwhelming and long-lasting injurious effects on target cells and organs and thereby contributing to diabetic complications. The risk for these complications increases with longer disease duration [2]. In diabetic rats, the alteration of glucose metabolism facilitates profound disturbances in lipid, amino acid and energy metabolism [7]. Therefore, detection of endogenous metabolic variations in the entire body as diabetes developing and progressing becomes highly significant.

NMR-based metabonomics, combines ^1^H nuclear magnetic resonance (^1^H NMR) spectroscopic profiling of biological samples with multivariate statistical analysis [8]. As such NMR-based metabonomics is well established as a powerful technique for rapidly identifying changes in the global metabolite profiles of biological samples [9], [10]. Given its usefulness in evaluating systemic responses to subtle metabolic perturbation, metabonomics has been used for the diagnosis and evaluation of diabetic patients [11] as well as the identification of its potential biomarkers [12], [13]. Diabetes can lead to pathological concentrations of plasma metabolites that are ultimately detected in urine [14], [15]. Korner et al. reported that urine composition in diabetes may result from the targeted effects of glucose on renal cells. These effects are evidenced by impaired solute reabsorption from the tubular lumen and reduced efficiency of the epithelium [14]. Williams et al. reported age-related changes in urinary metabolites over a five-month period by using normal Wistar-derived rats as the model. Urinary metabolic profiles were found to be correlated with the animal development [16]. We observed significant differences in the metabolic profiles between Zucker (fa/fa) obese and Wistar-derived rats at 12 weeks old and older than 4 weeks to 20 weeks [17]. We also found distinct differences in the composition of the urine and kidney tissue of male Sprague-Dawley rats at diabetic 2- and 8-wk periods [18]. In addition, we compared the urine of Zucker rats to Goto-Kakizaki rats. Hyperglycemia was shown to cause metabolic disorders, perturb the metabolic pathway in various organs, and induce metabolic imbalance. These effects eventually lead to functional deficits of the organs [19]. Nevertheless, a comprehensive understanding of metabolic characteristics during diabetes evolution remains to be achieved.

Metabolic data derived from urine samples can provide a systemic approach to investigating the detailed metabolic mechanisms of diabetes. We performed urinary NMR-based metabonomics analysis obtained from diabetic rats of 1, 5, 10, and 15 wks, as well as their age-matched controls. The purpose of this analysis was to extend the scope of our previous studies and further understand the pathogenesis of the onset and progression of diabetes on systemic and metabolic levels. The results of our study can facilitate the understanding of the complicated, potential molecular mechanisms of diabetes and its complications.

## Materials and Methods

### Animals

Male Sprague-Dawley rats with 160–180 g body weight were purchased from the Shanghai SLAC Laboratory Animal Co. Ltd., and kept in a SPF colony of Laboratory Animal Center of Wenzhou Medical College with regulated temperature and humidity and a 12/12 h light–dark cycle with lights on at 08:00 am. The animal production licenses are No. SCXK 2007-0005. During the whole experimental process, rats were fed with certified standard rat chow and tap water ad libitum.

### Ethics Statement

The protocol for the animal experiment was approved by the Institutional Animal Committee of Wenzhou Medical College. All animals received care in accordance to the ‘Guide for the Care and Use of Laboratory Animals’. Procedures using rats were approved by the Institutional Animal Care and Use Committee of Wenzhou Medical College (document number: wydw2012-0083).

### Experimental design and sample collection

After a 12 h fasting, some rats (n = 20) were randomly selected and injected intraperitoneally with streptozotocin (STZ, Sigma-Aldrich) freshly prepared in citrate buffer (0.1 M, pH 4.5) at a single dosage of 60 mg/kg body weight to prepare the diabetic model. And others (n = 16) were injected with the same volume of sodium citrate as the control. Two days after the injection, the blood glucose concentration was measured using a tail nick and glucometer (One Touch Ultra, Lifescan), by which the rats of blood glucose level higher than 16.70 mmol L^−1^ were defined as diabetic rats (n = 18). Each urine sample was collected for 12 h after 12 h fasting at the time points of 1, 5, 10, and 15 wks after STZ injection, respectively. The urine samples were collected over ice into 0.1 ml of 1% sodium azide solution and then centrifuged for 10 min at 4°C. The supernatant was stored at −80°C until measurement.

### Preparation of samples and acquisition of ^1^H NMR data

Before NMR analysis, urine samples were thawed, and 500 µL aliquots were mixed with 50 µL D_2_O containing TSP (0.36 mg mL^−1^) and 300 µL of phosphate buffer (0.2 M Na_2_HPO_4_/NaH_2_PO_4_, pH 7.4) to minimize variations in pH [20]. D_2_O provided a field-frequency lock, and TSP was used as the chemical shift reference. The mixtures were centrifuged to remove the precipitates, and then 500 µL of supernatant was transferred to 5-mm NMR tubes. All NMR spectra were recorded at 25°C on a Bruker AVANCE III 600 MHz NMR spectrometer equipped with a triple resonance probe and a z-axis pulsed field gradient. ^1^H NMR spectra were acquired using a one-dimensional NOESY pulse sequence with water suppression during the relaxation delay of 4 s and a mixing time of 150 ms. 128 free induction decays were collected into 32 K data points with a spectral width of 12000 Hz, an acquisition time of 2.66 s. FID was zero-filled to 64 K prior to Fourier transformation.

### Data reduction and multivariate pattern recognition analysis

All NMR spectra were phased and baseline corrected, and then data-reduced to 1000 integrated regions of 0.01 ppm width corresponding to the region of δ10 to 0 using the Topspin 2.1 software package for multivariate pattern recognition analysis. And another data-reduced to 6000 integrated regions of 0.0015 ppm width corresponding to the region of δ 8.98 to −0.02 for quantitative analysis. The region of the spectra associated with residual water and urea (4.63–5.05 and 5.54–6.0 ppm) were removed. And due to the presence of conspicuous glucose metabolite, the resonances region of glucose of all spectra were also excluded [21], [22]. The remaining spectral segments for each NMR spectrum were normalized to the total sum of the spectral intensity to partially compensate for differences in concentration of the samples. The pre-processed NMR data was then imported to SIMCA-P+ 12.0 software package (Umetrics, Umeå, Sweden) for analysis and visualization by multivariate statistical methods. Data was mean-centred and Pareto scaled prior to analysis for partial least squares-discriminant analysis (PLS-DA) and orthogonal-projection to latent structure- discriminant analysis (OPLS-DA). The advantage of OPLS-DA over PLS-DA is that the model incorporated an orthogonal signal correction algorithm and can identify two variances, one is correlates with the response and the other is orthogonal to the response. This separation of correlates and orthogonal components helps to the model interpretation. Metabolic trajectories were constructed with the average scores for each time point defining the path of the trajectory. Data were visualized with the scores plot of the two principal components (PC1 and PC2), in which each point represented an individual spectrum of a sample. The metabolites associated with the group separations were indicated by the corresponding loading plots, in which each point stood for a single NMR spectral region segment.

### Clinical chemistry measurements

Urine creatinine (CRE), urea nitrogen (UN) and uric acid (UA) were measured using automatic biochemical analyzer (Mindray BS-300). Values were expressed as the mean ± SD.

### Quantitative analysis

Statistical analysis was carried out using SPSS software (version 13.0, SPSS). Independent samples t test was applied in order to detect significant differences in selected signals between two groups. A P value of <0.05 was considered statistically significant.

## Results

### Biological traits

STZ-induced diabetic rats exhibited hyperphagia and polydipsia and consumed two to three times as much diet per day as did the control rats. Meanwhile, the urinary output of the STZ-diabetic group was significantly increased at all times, and the body weight decreased gradually after STZ treatment [23]. By 7 wks to 8 wks after STZ injection, the eyes of some diabetic rats developed lens opacity, which progressed into cataracts betweem 11 wks and 12 wks after injection. This finding was consistent with the previous report by Perry et al. [24].

### Clinical chemistry

UN, CRE and UA are metabolites of protein, creatine and purine, respectively. These metabolites are excreted with urine after glomerular filteration. Changes in UN CRE and UA levels in the urine directly reflect glomerular filtration. Clinical chemistry data (**Table 1**) revealed that both CRE and UA were significantly lower in the diabetic group than in the control group from 1 wk to 15 wks. However, BUN levels were higher in the diabetic 1-, 5-, and 9-wk groups, and lower in the diabetic 15-wk group, compared with their age-matched controls. These results indicated that renal function was significantly affected during the onset of diabetes, worsening with the development of the disease.

**Table 1:** Effect of STZ treatment on various urine parameters at different time points after induction.

	1-wk		5-wk		10-wk		15-wk	
	Con	DM	Con	DM	Con	DM	Con	DM
UA (mg/dL)	20.89±5.38	14.04±5.36**	10.96±2.81	8.19±1.57*	11.90±4.84	6.88±1.55*	9.07±2.31	6.97±1.32*
UN (mmol/L)	18.18±8.92	24.86±5.85*	14.15±3.10	19.65±5.65*	15.57±3.03	20.20±4.47*	21.47±4.77	13.47±3.4**
CRE (mg/dL)	82.17±43.38	54.60±14.47*	68.75±17.61	44.56±11.81**	79.86±19.37	40.47±9.35**	150.82±49.17	35.80±18.84**

Values are expressed as mean ± SD (n = 6 for each group). UA, uric acid; UN, urea nitrogen; CRE, creatinine.

* *p<*0.05,

** *p<*0.01 compared with the control group.

### NMR spectra and pattern recognition analysis of urine

**Figure 1:**
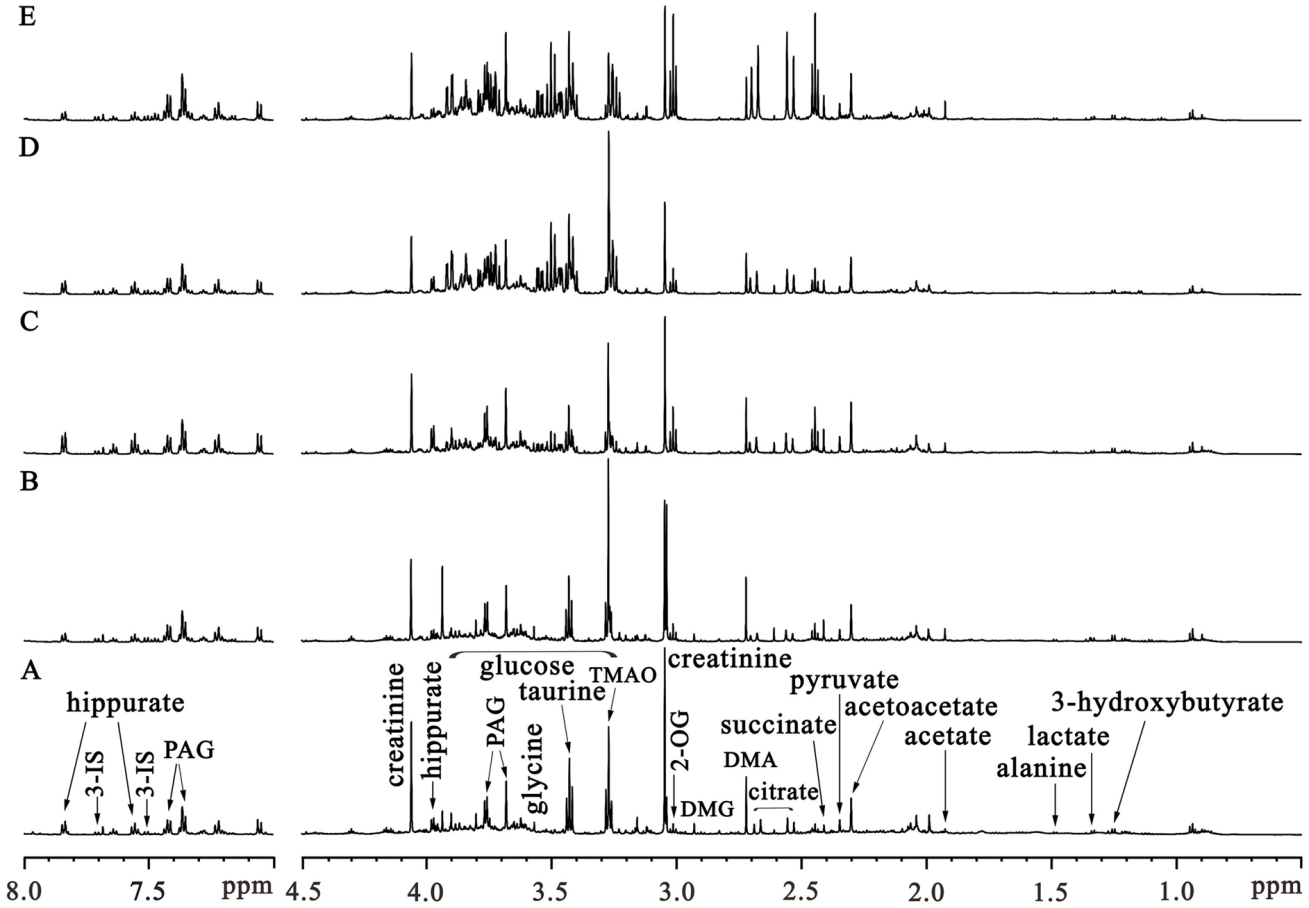
Representative ^1^H NMR spectra of urinary samples obtained from one control rat (A), and four rats at diabetic 1-wk (B), diabetic 5-wk (C), diabetic 10-wk (D), and diabetic 15-wk (E), respectively.

Representative ^1^H-NMR spectra of urine samples obtained from rats in the control and diabetic 1-, 5-, 10-, and 15-wk group are shown in **Figure 1A–E**, respectively. The spectral resonances of the metabolites were assigned according to our previous work [25] and the 600 MHz library of the Chenomx NMR suite 7.0 (Chenomx Inc., Edmonton, Canada). Compared with the control rats, the alterations of urinary profiles of diabetic rats were apparent in the NMR spectra following visual examination, with increased levels of glucose, acetoacetate and PAG, and decreased levels of creatinine.

**Figure 2:**
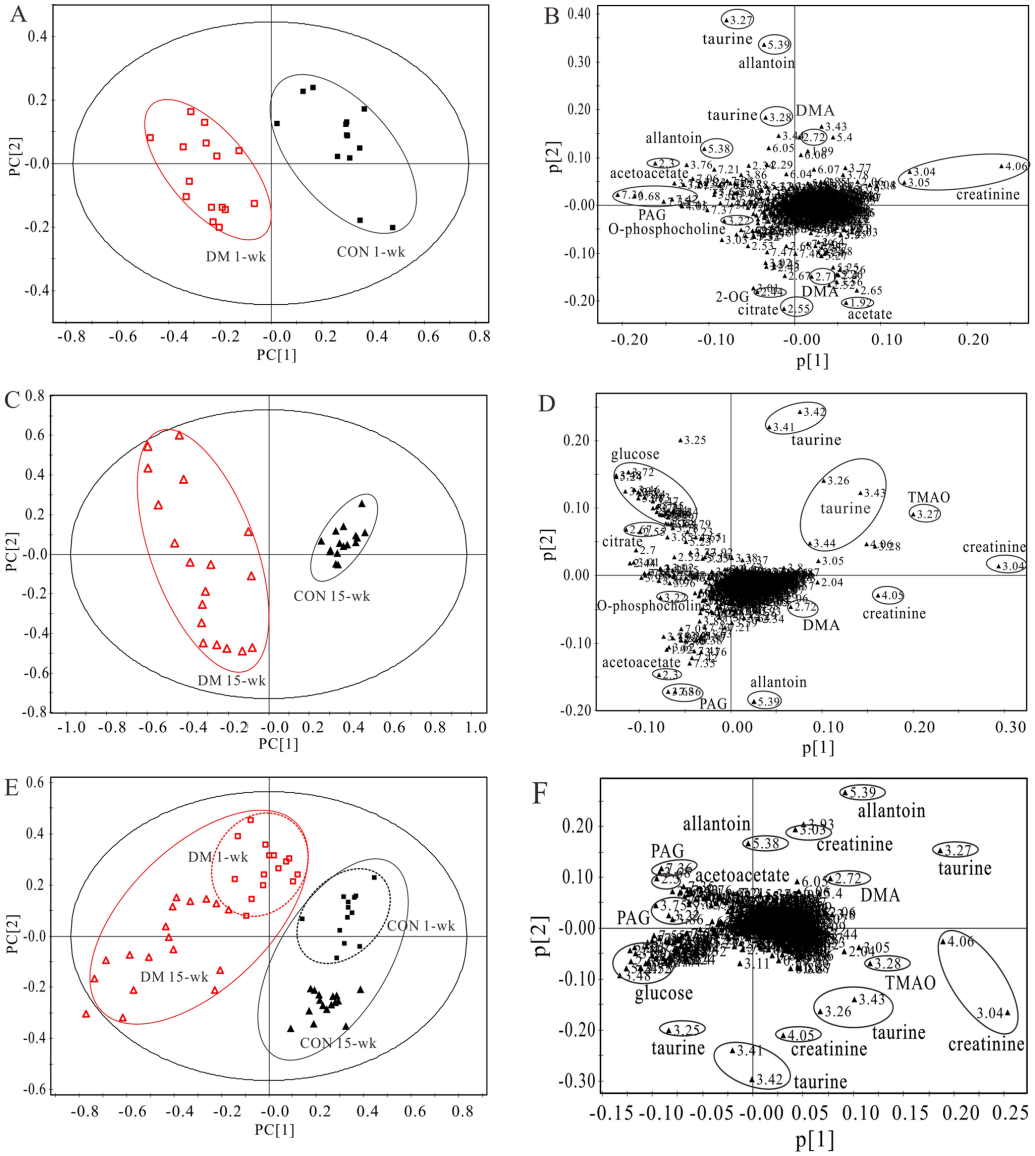
PLS-DA score plots based on ^1^H NMR spectra of urine samples from rats of (A) diabetic 1-wk (□, n = 15) and its control (▪, n = 13), (C) diabetic 15-wk (Δ, n = 18) and its control (▴, n = 16) rats, and (E) diabetic 1-wk, 15-wk, and their control rats, respectively. (B), (D) and (F) are the loading plots revealing the metabolites with large intensities responsible for the discrimination of the corresponding score plots (A), (C) and (E), respectively.

To explore the metabolic profiles of diabetes at different evolution stages, PLS-DA was performed based on the urine ^1^H NMR spectra of the diabetic 1-, and 15-wk rats as well as their age-matched controls (**Figure 2**). As shown in **Figure 2A**, clear discrimination along the PC1 direction was observed between diabetic 1-wk rats and their age-matched controls (R^2^X = 0.910, Q^2^Y = 0.850). The corresponding loading plot (**Figure 2B**) revealed that the diabetic 1-wk rats excreted lower level of creatinine, but higher levels of PAG, acetoacetate, ascorbate, allantoin, 2-OG, o-phosphocholine and citrate than their controls. Obvious urine metabonomics differentiation is also found in the diabetic 15-wk rats and their age-matched controls (**Figure 2C**, R^2^X = 0.934, Q^2^Y = 0.913). The corresponding loading plot (**Figure 2D**) illustrated that the urine of diabetic 15-wk rats had decreased levels of creatinine, creatine, taurine, TMAO and allantoin, but increased levels of glucose, hippurate, acetoacetate, citrate, 2-OG, PAG, acetate and o-phosphocholine. The PLS-DA scores plots and loading plots for the spectra of the 5- and 10-wk urine samples showed similar changes.

The PLS-DA scores plot shows diabetic 1- and 15-wk groups as well as their age-matched controls clustered together, respectively (**Figure 2E**, R^2^X = 0.556, Q^2^Y = 0.533), and the diabetic groups are clearly separated from their age-matched controls along the PC1 direction. What is more, the diabetic 1-wk group and diabetic 15-wk group are separated along the PC2 direction, as well as that in the control groups. The diabetic 15-wk group is more dispersed than the diabetic 1-wk group, which came out the multiple dispersions of diabetic complications as we had expected. And the corresponding loading plot (**Figure 2F**) suggests that the separation was attributed to the variables, including creatinine, TMAO, taurine, allantoin, PAG, DMA, acetoacetate and glucose.

**Figure 3:**
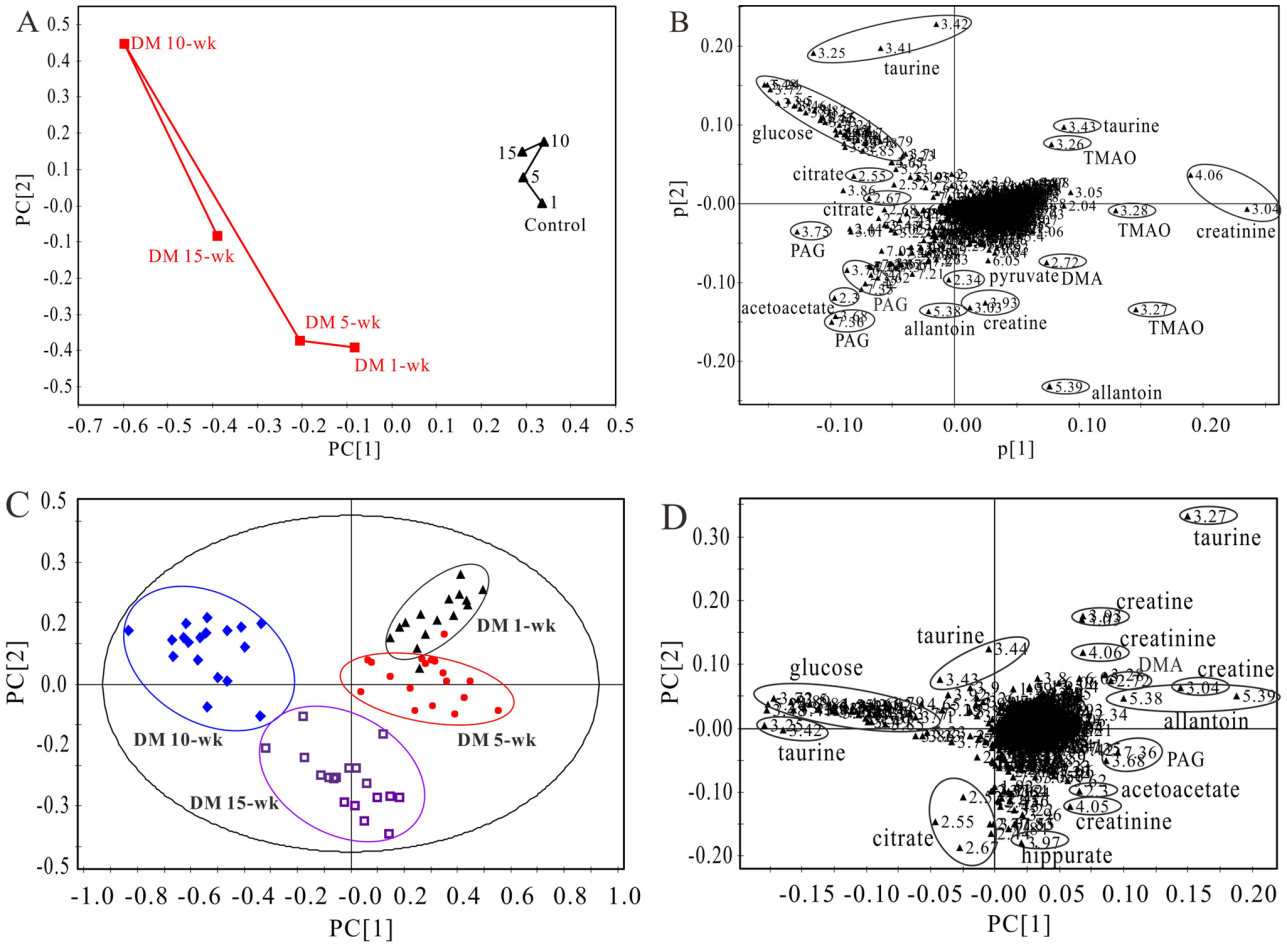
(A) PLS trajectory based on the mean ^1^H NMR spectra of urine samples collected from animals treated with STZ (▪) at various time points (1-wk, 5-wk, 10-wk, and 15-wk), and the age-matched control rats (▴). (C) OPLS-DA scores plot based on ^1^H NMR spectra of urine samples from diabetic 1-wk (▴, n = 15), 5-wk (•, n = 17), 10-wk (♦, n = 18), and 15-wk (**□**, n = 18) rats. (B) and (D) are the loading plots revealing the metabolites with large intensities responsible for the discrimination of the corresponding score plot shown in (A) and (C), respectively.

To characterize completely the metabolic changes that occur at different stages of diabetes evolution, the PLS-DA mean trajectory was employed based on the urine ^1^H NMR spectra of diabetic 1-, 5-, 10-, and 15-wk rats as well as their age-matched controls, which exhibits a distinct separation between diabetic group and the control group along the PC1 direction (**Figure 3A**, R^2 = ^0.985, Q^2 = ^0.956), The diabetic groups showed a rapid change during the period of 1 wk to 15 wks, while the control groups were found to occupy a relatively similar position in the “metabolic space”, probably because the influence of diabetes progression on the metabolic pattern is stronger than that of the growth and development of the rats themselves. The corresponding loading plot (**Figure 3B**) indicates that the variables corresponding to allantoin, TMAO, creatinine, taurine, hippurate, PAG, DMA, acetoacetate, citrate, pyruvate, 3-Indoxylsulfate (3-IS) and glucose were the most responsible for the separation. It is further demonstrated by the OPLS-DA scores plot obtained from the urine of diabetic rats at four time points, where diabetic rats are separated from each other along the PC1 direction (**Figure 3C**, R^2 = ^0.863, Q^2 = ^0.650). An extended interval after STZ administration results in improved discrimination of the diabetic group cluster from the diabetic 1-wk cluster. Instead, the metabolic pattern of diabetic 15-wk rats is close to that of the diabetic 5-wk rats, which indicates the regression of metabolic pattern when diabetes developed to complications. The corresponding loading plot shows that the variables that referred to allantoin, TMAO, creatine, hippurate, PAG, DMA, acetoacetate, citrate and glucose were the most responsible for the separation (**Figure 3D**).

### Changes in metabolite concentrations

**Figure 4:**
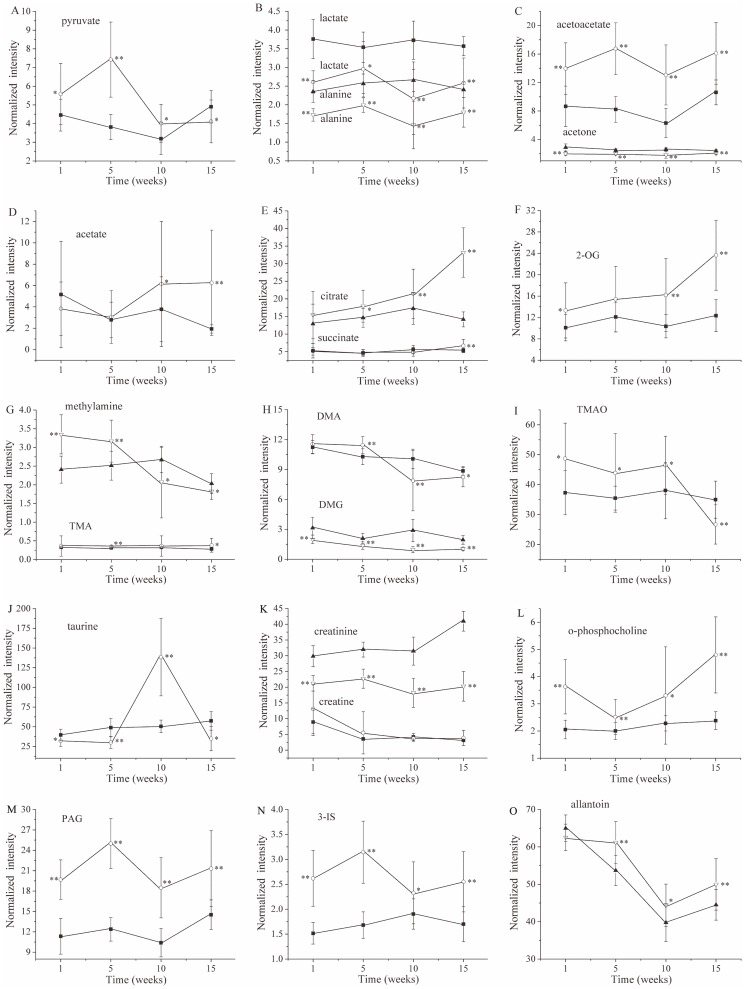
Metabolic alterations obtained from ^1^H NMR spectra of urine samples collected from control group (shaded markers, n = 13–16) and diabetic group (open markers, n = 15–18) rats of 1 wk, 5 wks, 10 wks, and 15 wks after STZ injection. The results are expressed as means ± standard deviations. ^*^p<0.05 and ^**^p<0.01 compared to age-matched control rats by independent samples t-test.

In order to make the metabolic changes picked up by PLS-DA and OPLS-DA clearer, the excreted levels of metabolites were compared between the diabetic groups and their controls on the basis of the ^1^H NMR peak intensities. The key metabolites that were associated with certain metabolic pathways and changed over time during diabetes onset and progressions are shown in **Figure 4**. Pyruvate produced by glucose anaerobic metabolism increased during the onset of diabetes (1 and 5 wks) and decreased during diabetes evolution (10 and 15 wks). Two additional glycolysis-related products, alanine and lactate, indicated significantly decreased levels during the entire experimental period (**Figure 4A–B**). Acetoacetate and acetone comprise ketone bodies, which are metabolic products of fatty acids in liver mitochondria. Acetoacetate increased, but acetone decreased. Acetate is another product of fatty acid β-oxidation, which remained at a constant level during diabetes onset and increased during diabetes evolution (**Figure 4C–D**). As important tricarboxylic acid (TCA) cycle intermediates, the levels of citrate and 2-OG increased and reached the maximum at diabetic 15-wk group. The level of succinate almost remained the same at 1-, 5-, and 10-wk diabetic group; however, it significantly increased at 15 wks (**Figure 4E–F**). Methylamine, DMA, TMA, DMG, TMAO and betaine are related to methylamine metabolism. At diabetic 5 wk, the levels of methylamine, DMA, and TMA increased, and then they decreased at diabetic 10 and 15 wk. TMAO excretion was enhanced in the diabetic 10-wk group but was reversed in the diabetic 15-wk group. The change in betaine was opposite to the changes in DMA, and DMG, which was decreased in the diabetic group at all studied time points (**Figure 4G–I**). Taurine was found to be less excreted in diabetic 1-, 5-, and 15-wk rats and more excreted in 10 wk in the diabetic rats. Creatinine displayed decreased in diabetic group from 1 wk to 15 wks. O-phosphocholine, PAG, 3-IS and allantoin showed similar increasing trends in diabetic rats (**Figure 4J–O**).

## Discussion

Type 1 diabetes mellitus is an autoimmune disease that results in permanent destruction of the insulin-producing cells in the pancreas and subsequent disturbance of glucose and lipid metabolism. This persistent metabolic imbalance is linked to high incidence of vascular complications [19]. In this study, the ^1^H NMR spectra of urine samples from the 1-, 5-, 10-, and 15-wk diabetic rats after STZ induction indicated marked changes in the levels of key endogenous metabolites compared with the age-matched controls. This result can provide in detail the pathogenesis of the development and progression of diabetes and help in the early diagnosis and prevention of diabetes and its complications.

There are several reports about diabetes using ^1^H NMR, HPLC-MS or GC-MS to determine the changes in metabolite profiles of tissue [26] and biofluids [21], [22]. These studies are of limited value in indentifying biomarkers of disease-related profiles for monitoring disease onset and development. In this study, the OPLS-DA results indicate that the metabolic profiles of diabetic 1-wk samples are closer than those of other time points, which may be due to hyperglycemia resulting from decreased glucose metabolism and augmented glucose accumulation at the acute stage of diabetes. The dispersed metabolic distribution of diabetic 10 and 15-wk urine samples may result from various diabetes complications, such as diabetic nephropathy [18], [27], liver disease [28] and diabetic retinopathy [24] during the diabetes evolution. There are some works demonstrated that hyperglycemia caused metabolic disorder and perturbed metabolic pathways in various organs, and eventually leaded to the functional deficits of the organs in diabetic rats [29]. We have reported that the metabolic profiles of liver was altered at the early stage of diabetic rats [19], and 8-wk diabetic rats showed obvious diabetic nephropathy [18]. These works are consistent with the results of the present study.

**Figure 5:**
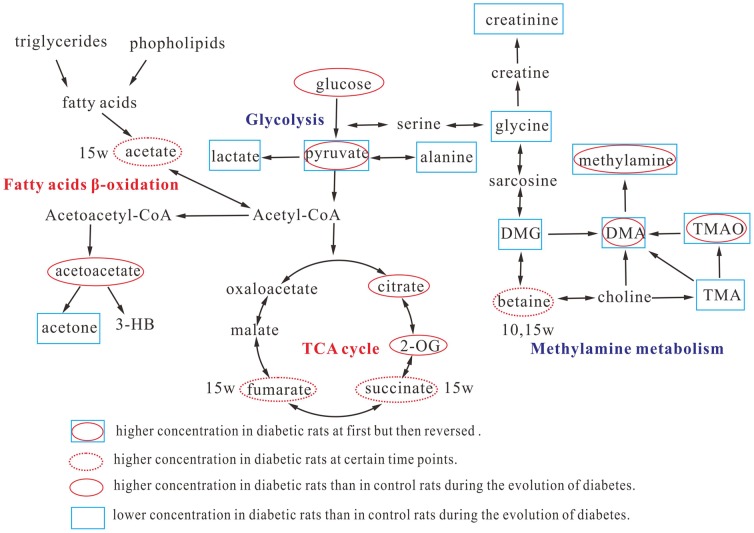
Schematic diagram of the most relevant metabolic differences between diabetic rats and their age-matched control rats during the evolution of diabetes.

The variations of metabolites allowed us to obtain some important metabolic information about the mechanisms involved in the evolution of diabetes. Actually, systemic abnormalities happened in diabetic rats can lead to pathological changes of several metabolites in plasma and consequently, to the urinary overflow. The altered metabolite levels indicate significant metabolic changes in the glucose metabolism and the TCA cycle in diabetic rats. **Figure 5** illustrates the metabolic pathways based on the KEGG database (http://www.genome.jp/kegg/pathway.html) involved in the evolution of diabetes.

The energy needed for the physical function requires ATP, which primarily originates from glycolysis, glucose oxidation and lipid oxidation. Diabetic animals lack insulin or cannot effectively use insulin, leading to impaired glucose uptake and utilization [19]. Following STZ injection, enhanced endogenous glucose from noncarbohydrate precursors and glycogen breakdown causing hyperglycemia have been observed [30]. In urine samples of diabetic rats, a number of metabolites involved in energy metabolism are perturbed because of hyperglycemia and hypoinsulinemia. Citrate and 2-OG are the intermediates of the TCA cycle and are essential in energy metabolism. Pyruvate is an important product of glycolysis and can be converted into acetyl-CoA, which then enters the TCA cycle. In this work, increased levels of pyruvate, 2-OG and citrate, as well as decreased level of lactate in diabetic 1-, 5-, 10-wk rats are in good agreement with the results of a previous study. The previous result indicated that the increases in relative concentrations of the TCA cycle intermediates of citrate and fumarate were observed in *fa/fa* rats and *db/db* mice [21]. This finding suggests that the pathways of glycolysis are inhibited and that aerobic metabolism is promoted in the development of diabetes. This result is also consistent with our recent finding that increased excretion of TCA cycle intermediates was observed in the urine of diabetic 2- and 8-wk rats [18]. As diabetic rats have glycosuria, the increases in urinary concentration of TCA cycle intermediates may reflect either systemic stress produced by hyperglycemia or local effects on kidney tubular transport [21]. At the diabetic 15-wk period, the increased levels of 2-OG, citrate and succinate as well as decreased levels of lactate and pyruvate are clearly exhibited. These changes suggest the inhibition of glycolysis and the enhancement of aerobic metabolism. Thus, enhanced TCA cycle results from increased catabolism of amino acid at the late stage of diabetes.

Acetate is an end product of fatty acid oxidation, and the increased acetate in diabetic 10- and 15-wk rats indicates an increased acetyl-coenzyme A (CoA) pool as a result of production by both exogenous (bacterial fermentation) and endogenous (mammalian) [31], [32]. The increased acetate has been reported in the urine of patients with type 2 diabetes for the impaired renal function [33]. What is more, the increased levels of acetoacetate and acetate from diabetic 1- to 15-wk suggest the enhanced β-oxidation pathway of fatty acids during the onset and development of diabetes, which may result in the increased concentration of the intermediates of TCA. This hypothesis was supported by the report of Rennie that the increased provision of free fatty acids caused an increase in free fatty acid oxidation [34].

The fluctuation of methylamines in the urine of diabetic rats is indicative of a change in the balance of methylamine metabolism. Methylamines can be derived from dietary choline, which break down to monoamine, DMA and TMA by gut microflora [35]. And polyamines are produced systemically by degradation of choline to betaine and DMG [21]. One of the key roles of methylamines is to act as osmoregulatory compounds [36]. The increased levels of TMA, DMA, TMAO and methylamine and decreased level of DMG shown in diabetic 1- and 5-wk rats suggest an increased mammalian uptake of dietary choline. Alternatively, an attenuated conversion of choline into DMG may occur, as compared with its conversions to DMA at the early stage of diabetes. Upregulation of the methylamine pathway in vivo may be linked to the hyperosmotic effect of glucose, or indicate renal papillary dysfunction [36]. At diabetic 10-wk period, the increased DMA and methylamine were reversed. However, TMAO and TMA continued to increase, and DMG continued to decrease, which imply the potentially attenuated conversion of choline to DMG and the conversions of choline to DMA, leading to decreased methylamine in the development of diabetes. At diabetic 15-wk, the increased TMAO was reversed, which may be responsible for the further reduced methylamine at the late stage of diabetes. More studies are required to understand in detail the reasons for the switch in the correlations among the metabolites of the choline pathway.

The betaine excretion was increased at diabetic 10 and 15 wks, indicating abnormal renal functions as evidenced by microalbuminuria and tubular function markers [33]. The hypothesis was further supported by apparent signs of urine protein in the ^1^H NMR spectra and the results of urine biochemical assay. Another osmolyte, o-phosphocholine, demonstrated an increasing trend from diabetic 1 wk to 15 wks. This change may reflect the systemic protective response to the stimulation of hyperglycemia.

Taurine is the most abundant free amino acid present in many tissues. In addition to its benefit for liver dysfunction indication, taurine is also essential in osmoregulation by regulating cell volume [37]. In the diabetic states, elevated extracellular glucose levels apparently represent osmotic stress for the cell. Production or transport of additional intracellular osmolytes are needed to maintain osmotic balance across the cell membrane [38]. This is consistent with the increase in taurine in the urine of diabetic 10-wk rats observed in the present study. Urinary taurine concentration is primarily regulated by renal reabsorption, whereas reabsorption is consequently regulated by taurine availability [21]. An increase in taurine level may arise from a reduced glomerular filtration rate, leading to altered renal reabsorption of taurine, particularly following damage to the liver [39].

Hippurate is normally found in urine, and its concentration is related to the microbial activity and composition of the gut [10]. Furthermore, urinary hippurate has been reported to be an early biomarker of nephrotoxicity resulting from nephrotoxin administration in rats [40]. Changes in kidney function can be expected to be reflected in urinary composition [16]. An increased level of hippurate in the urine of diabetic 15-wk rats indicates an alteration in gut microbiota. Allantoin represents a convenient biomarker of oxidative stress, and it was increased in the plasma of type 1 diabetic patients. This increase is attributed to the increase in the rate of superoxide production resulting decreased mitochondrial phosphorylation activity [41]. In this study, the elevation of allantoin in the urine of diabetic 5-, 10- and 15-wk rats indicated an increased glomerular filtration rate, given that allantoin is not reabsorbed across the proximal tubule [42]. Longer periods after STZ injection caused a stronger peak signal of glucose and protein in urine in diabetic rats. This observation indicates that kidney function was affected during the evolution of diabetes.

Creatinine is an indicator of glomerular filtration rates, and exclusively formed from creatine in the body [43]. This by-product exhibited a decreasing trend in the urine of diabetic rats from 1 wk to 15 wks, compared with the control. This result is consistent with that in a previous work [21] and may been caused by changes in muscle mass, creatinine reabsorption, cell leakage, and caloric intake. Meanwhile, PAG is generated from phenylacetate in the liver or the gut mucosa [43]. The increased PAG excretion in the diabetic rats from 1 wk to 15 wks in this study suggests changes in intestinal microflora during the onset and development of diabetes.

It should be noted that the exact mechanisms leading to the observed metabolic changes should be further studied. A limitation of this study is that the rates of metabolism or cycling of a specific site in metabolic pathways that may be disrupted in the diabetes progress were not provided, for the ^1^H NMR measures used only suggest a static picture of metabolites measured. Future explorations using ^13^C NMR measures of labeled substrates incorporation into the metabolites of diabetic rats are likely to provide additional information regarding the specific mechanisms about the observed abnormalities of the metabolites.

## Conclusion

In summary, our metabonomic analyses of urine from diabetic 1-, 5-, 10-, and 15-wk rats highlighted characteristic metabolic features during the onset and progression of diabetes. Systemic perturbations in endogenous metabolites may provide clues that can elucidate the mechanisms underlying the generation and development of diabetes and its complication. Altered metabolic pathways including enhanced β-oxidation of fatty acids and inhibited glycolysis were observed in diabetic rats. The methylamine pathway was also up-regulated at the early stage but downregulated with the development of diabetes as found in this study. More NMR-based metabonomics studies involving long-term studies of large numbers of diabetic animal models and patients are expected to associate these results with clinical indicators of urine and blood tests. Further investigation is also expected to provide comprehensive knowledge of diabetes and its complications at the molecular level.
